# High-Content Imaging Reveals Expansion of the Endosomal Compartment during *Coxiella burnetii* Parasitophorous Vacuole Maturation

**DOI:** 10.3389/fcimb.2017.00048

**Published:** 2017-02-28

**Authors:** Charles L. Larson, Robert A. Heinzen

**Affiliations:** Coxiella Pathogenesis Section, Laboratory of Bacteriology, Rocky Mountain Laboratories, National Institute of Allergy and Infectious Diseases, National Institutes of HealthHamilton, MT, USA

**Keywords:** endosome, lysosome, intracellular pathogen, endocytic trafficking, fluorescence microscopy, *Chlamydia trachomatis*, *Coxiella burnetii*

## Abstract

*Coxiella burnetii* is an obligate intracellular pathogen and the causative agent of human Q fever. Replication of the bacterium within a large parasitophorous vacuole (PV) resembling a host phagolysosome is required for pathogenesis. PV biogenesis is a pathogen driven process that requires engagement of several host cell vesicular trafficking pathways to acquire vacuole components. The goal of this study was to determine if infection by *C*. *burnetii* modulates endolysosomal flux to potentially benefit PV formation. HeLa cells, infected with *C. burnetii* or left uninfected, were incubated with fluorescent transferrin (Tf) for 0–30 min, and the amount of Tf internalized by cells quantitated by high-content imaging. At 3 and 5 days, but not 1 day post-infection, the maximal amounts of fluorescent Tf internalized by infected cells were significantly greater than uninfected cells. The rates of Tf uptake and recycling were the same for infected and uninfected cells; however, residual Tf persisted in EEA.1 positive compartments adjacent to large PV after 30 min of recycling in the absence of labeled Tf. On average, *C. burnetii*-infected cells contained significantly more CD63-positive endosomes than uninfected cells. In contrast, cells containing large vacuoles generated by *Chlamydia trachomatis* exhibited increased rates of Tf internalization without increased CD63 expression. Our results suggest that *C. burnetii* infection expands the endosomal system to increase capacity for endocytic material. Furthermore, this study demonstrates the power of high-content imaging for measurement of cellular responses to infection by intracellular pathogens.

## Introduction

*Coxiella burnetii* is an intracellular bacterial pathogen capable of infecting a broad range of host organisms. Symptomatic infection of humans, called Q fever, normally manifests as an acute flu-like disease with fever and body aches that can last several weeks. Less frequently, chronic endocarditis or hepatitis occurs (Eldin et al., 2017). Aerosolically-transmitted *C. burnetii* has a tropism for mononuclear phagoctyes with the pathogen initially targeting alveolar macrophages (Stein et al., 2005).

Following internalization of *C. burnetii* by host cells, the nascent pathogen-occupied vacuole matures through the endolysosomal pathway to acquire characteristics of a phagolysosome (Romano et al., 2007; Howe et al., 2010). The limiting membrane of the mature parasitophorous vacuole (PV) decorates with lysosome-associated membrane proteins (LAMPs), and the PV lumen acidifies and acquires active cathepsins (Howe et al., 2010). Concurrent with vacuole acidification is *C. burnetii* metabolic activation and translocation of effector proteins into the host cell via a Dot/Icm type 4B secretion system (T4BSS) (Newton et al., 2013). T4BSS effector proteins modify the PV into a replication-permissive niche by subverting several host cell functions including those involved in vesicular trafficking (Beare et al., 2011; Carey et al., 2011; Larson et al., 2016). The *C. burnetii* PV can occupy nearly the entire host cell cytoplasm. Accordingly, expansion of the nascent vacuole into a large replication-permissive niche is predicted to require substantial manipulation of the endosomal system.

Plasma membrane endocytosis is a continual process in mammalian cells (Huotari and Helenius, 2011). Degradation of internalized cargo within the endosomal pathway begins with vesicles generated by endocytosis and ends with lysosomal fusion (Saftig and Klumperman, 2009; Huotari and Helenius, 2011). Endocytic vesicles fuse with peripherally localized early endosomes (EEs) where the endocytosed material is sorted and either recycled out of the cell or transported further within the endosomal system. Fusion between EEs and new endocytic vesicles ceases after ~10 min as EEs undergo default maturation and become increasingly acidified (Maxfield and McGraw, 2004; Huotari and Helenius, 2011). Maturing endosomes also translocate toward the center of the cell, transitioning into perinuclear recycling endosomes where additional recycling away of lipids and proteins occurs prior to delivery of remaining cargo to late endosomes (LEs) or multivesicular bodies. In the final step of maturation, the lumenal contents of late endosomal vesicles are degraded by acid hydrolases delivered by lysosomal fusion (Huotari and Helenius, 2011).

Several membrane trafficking pathways participate in PV formation and pathogen growth. Defects in *C. burnetii* replication and PV expansion occur when endosomal trafficking is suppressed by inhibition of Rab5 or Rab7 (Romano et al., 2007; McDonough et al., 2013), or disruption of endolysosomal fusion proteins syntaxin-17 (McDonough et al., 2013) or VAMP7 (Campoy et al., 2013). Dysregulation of Rab1 (Campoy et al., 2011) and Rab24 (Gutierrez et al., 2005), key regulators of secretory and autophagic systems, respectively, also impairs *C. burnetii* growth in host cells. Furthermore, impaired homotypic fusion of the PV has been linked to subversion of the autophagic system by the *C. burnetii* effector CvpB/Cig2 via a mechanism involving phosphoinositide manipulation (Newton et al., 2014; Kohler et al., 2016; Martinez et al., 2016). Indeed, *Coxiella* vacuolar proteins (Cvp) are a group of Dot/Icm effectors that, when ectopically expressed in *C. burnetii*-infected cells, localize to the limiting membrane of the PV. *C. burnetii* mutants in *cvpA, -B, -C, -D*, and *-E* all exhibit defects in replication and PV formation (Larson et al., 2013, 2015; Martinez et al., 2014, 2016; Newton et al., 2014). When ectopically expressed, CvpA localizes to vesicles that label with the early/recycling endosome marker transferrin (Tf) receptor (Larson et al., 2013, 2015). Tf co-localization coincides with CvpA interactions with clathrin and the tetrameric clathrin adaptor protein complex AP2. The formation of clathrin-AP2 coats on the luminal surface of the plasma membrane triggers vesicle budding and endocytosis of many proteins implicated in PV biogenesis, including LAMP1, Tf receptor, and mannose-6-phosphate receptor (Braulke and Bonifacino, 2009; Saftig and Klumperman, 2009). The effector protein Cig57 also co-opts clathrin-mediated trafficking by interacting with the clathrin-coated pit accessory protein FCHO2 (Latomanski et al., 2016).

The *C. burnetii* PV phenotypically resembles a large, temporally stable phagolysosome. Endocytosis and endocytic recycling play key roles in endosomal compartment structure and function. Consequently, manipulation of endocytic trafficking is predicted to be an important strategy employed by *C. burnetii* to promote PV biogenesis. The objective of this study was to determine whether *C. burnetii* infection of host cells alters normal endocytic trafficking. Quantitative light microscopy coupled with high-content image analysis was conducted to measure endocytosis and recycling of fluorescently labeled Tf, a marker commonly used to track endocytic trafficking (Hirschmann et al., 2015). Cells infected with *C. burnetii* exhibited greater amounts of internalized Tf, with the largest increases occurring in cells with mature PV. This behavior was associated with a general expansion of the endocytic compartment. These data provide new details on manipulation of vesicular trafficking by *C. burnetii* to promote infection.

## Materials and methods

### Cultivation of the bacteria

*C. burnetii Nine* Mile phase II (clone 4, RSA439) was propagated in Vero (African green monkey kidney) cells (CCL-81; American Type Culture Collection [ATCC]) and *Chlamydia trachomatis* LGV-434, serotype L2, was propagated in HeLa 229 cells (CCL-2; ATCC). Bacteria were purified from host cells as previously described (Caldwell et al., 1981; Shannon and Heinzen, 2007). *C. burnetii* stocks were enumerated by measuring genome equivalents (GE) by quantitative PCR using primer/probe sets specific to the *dotA* gene (Howe et al., 2010). *C. trachomatis* stocks were enumerated by measuring inclusion-forming units as previously described (Moore et al., 2008).

### Cell infection

Infected and uninfected HeLa cells were cultured in Dulbecco's Modified Eagle Medium (DMEM) (Thermo Fisher Scientific, Rockville, MD) containing 10% fetal bovine serum (FBS) and incubated at 37°C in 5% CO_2_. Semi-confluent cell monolayers were trypsinized and 2.5 × 10^5^ cells were seeded in T25 cell culture flasks (Corning, Corning, NY). Cells were infected with *C. burnetii* at a multiplicity of infection (MOI) of 50 based on GE, or left uninfected. The day before conducting Tf assays, matched flasks of infected or uninfected HeLa cells were trypsinized and 6 × 10^3^ cells per well seeded in 96-well plastic bottom plates (Ibidi, Martinsried, Germany). For infections with *C. trachomatis*, 6 × 10^3^ HeLa cells per well seeded in 96-well plastic bottom plates were infected at a MOI of 100 and incubated for 26 h. Infection conditions resulted in approximately 80% of cells infected with *C. burnetii* or *C. trachomatis*.

### Tf internalization and recycling assays

Measurement of cellular Tf internalization and recycling was assessed using methods previously validated by Hirschmann et al. (2015) with several modifications. Cells in 96-well plates were washed once with phosphate buffered saline (PBS; 12.8 mM KH_2_PO_4_, 72.6 mM NaCl, 53.9 mM Na_2_HPO_4_, pH 7.4) and then serum starved for 30 min in 150 μl assay medium (DMEM, 1% BSA, 20 mM HEPES, pH 7.2) at 37°C and 5% CO_2_. Alexa Fluor 488-labeled transferrin (Tf488) (Thermo Fisher) was prepared according to the manufacturer's instructions and diluted in assay medium to 125 μg/ml. For Tf internalization, 96-well plates were placed in a 37°C water bath, and Tf internalization was initiated by aspirating wells and adding 100 μl of pre-warmed 37°C Tf488 suspension. All samples were assayed in triplicate wells and separate sample wells were left untreated (no Tf488) to control for background signal from cell autofluorescence. To halt uptake of Tf488, plates were placed on ice and immediately washed once with ice-cold PBS, once with ice-cold acid wash buffer (0.5% acetate, 0.5 M NaCl, pH 3.0) to remove remaining surface-bound probe, and then three times with PBS. Cell cultures used to measure Tf internalization were fixed with 4% paraformaldehyde in PBS for 30 min whereas cultures used to measure Tf recycling were placed back in the 37°C water bath and incubated with 100 μl of pre-warmed assay buffer containing 50 μM deferoxamine mesylate (Sigma) to prevent re-uptake of recycled Tf. Recycling was stopped with the addition of 4% paraformaldehyde. To prepare samples for imaging, wells were washed 3 times with PBS, blocked for 10 min with PBS containing 5% BSA, and permeabilized with PBS containing 0.1% Triton X-100 and 5% FBS for 5 min. The bacteria were immunostained with rabbit anti-*C. burnetii* or rabbit anti-*C. trachomatis* serum, and Alexa Fluor 594-conjugated donkey anti-rabbit secondary antibody (Thermo Fisher). Nucleic acids were stained with 2 μM Hoescht 33342 (Thermo Fisher) and cells were stained with HCS CellMask™ Deep Red (Thermo Fisher) whole cell stain (WCS) in PBS according to the manufacturer's specifications.

### Image acquisition and analysis

Automated image acquisition with Zeiss Zen imaging software was used to collect 2 × 2 tiled images (2048 × 2048 pixels) of triplicate sample wells using a Carl Zeiss LSM 710 laser scanning microscope equipped with a Plan-APOCHROMAT 20 × /0.8 objective. Imaged fields were selected at random and acquired using autofocus mode with the pinhole completely open to simulate wide-field acquisition. A CellProfiler image analysis pipeline was designed for image segmentation, sorting of infected and uninfected cells, and measurement of signal intensities in each cell. The Hoescht (blue), Tf488 (green), *C. burnetii* (red), and WCS (far red) channels captured for each image were imported as gray-scale Tiffs into CellProfiler for analysis.

### Data analysis

CellProfiler data were exported as spreadsheets for analysis. Tf488 and *C. burnetii* per cell measurements were extracted with Microsoft Excel 2013 and imported into GraphPad Prism 7.01 for removal of outliers (ROUT, Q = 1%) and to perform statistical calculations. The mean Tf488 integrated intensity per cell was used to compare amounts of internalized Tf488 in each sample after subtracting background signal due to autofluorescence in the green channel. Background fluorescence signal in the absence of Tf488 was calculated using matched cells and cell cultures (i.e., uninfected, infected, or cells containing large PV or inclusions) at the designated time points. Statistical significance was determined by two-way ANOVA using Tukey's test for multiple comparisons.

### Immunofluorescence microscopy

For immunostaining of cellular markers, HeLa cells were fixed and permeabilized as described above, then incubated with primary antibodies against CD63 (BD Biosciences 556019), Tf receptor (ThermoFisher 136800), or EEA.1 (BD Biosciences 610456). Primary antibodies were detected with Alexa Fluor 568 conjugated donkey anti-mouse antibody (Thermo Fisher). A Carl Zeiss LSM 880 laser scanning microscope equipped with a Plan-APOCHROMAT 63 × /1.4 objective and Airyscan detector was used to capture super-resolution images of infected cells. For quantification of surface-localized Tf receptor, samples were fixed with 4% paraformaldehyde for 30 min, blocked 15 min with PBS plus 1% BSA, and incubated 45 min with 1:250 dilution of antibody against Tf receptor (ThermoFisher A11130) in PBS with 1% BSA. Image acquisition and quantitation of Tf receptor and CD63 were conducted as described for measurement of Tf488. Background signal was normalized using cells stained with anti-mouse Alexa Fluor 568 in the absence of primary antibody.

## Results

### Tf488 traffics to puncta adjacent to the *C. burnetii* PV.

**Figure 1:**
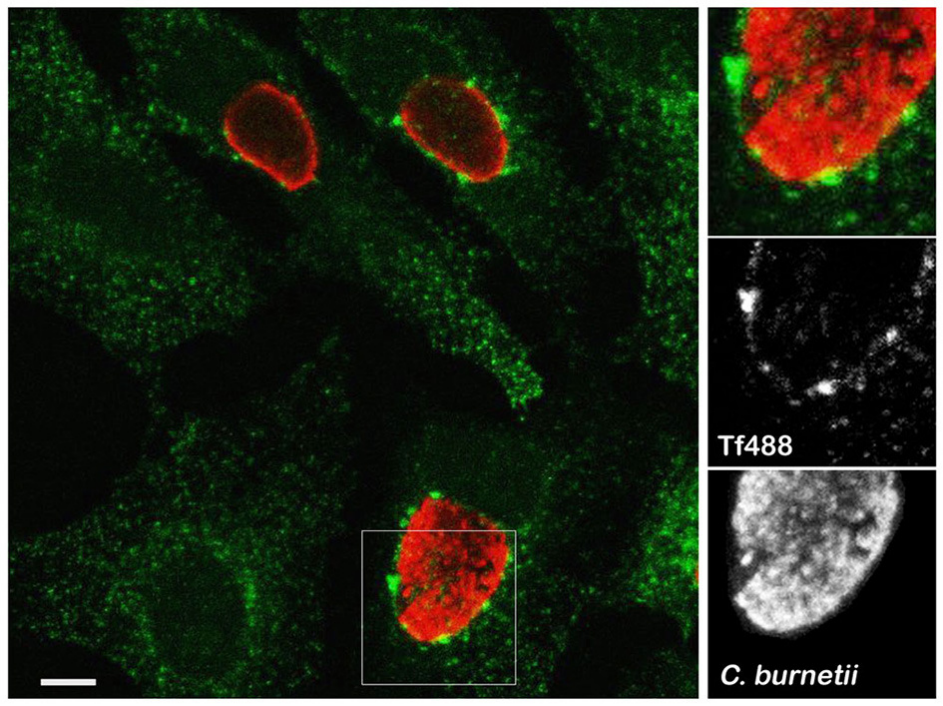
**Large Tf puncta localize adjacent to the *C. burnetii* PV**. Representative confocal fluorescence micrograph depicting Tf488 localization in HeLa cells infected for 3 days with *C. burnetii*. Cells incubated with Tf488 (green) for 30 min were fixed and stained with a rabbit anti-*C. burnetii* serum and Alexa Fluor 594 conjugated anti-rabbit secondary antibody (red). Scale bar, 10 μm.

Clathrin accessory proteins recognize Fe^3+^-loaded Tf bound to Tf receptor on the plasma membrane and sequester the complex into clathrin-coated pits that bud and form endocytic vesicles (Maxfield and McGraw, 2004; Braulke and Bonifacino, 2009). Internalized vesicles harboring the Tf complex fuse with early endosomes where Fe^3+^ is released and Tf and Tf receptor are recycled to the cell surface (Maxfield and McGraw, 2004; Hirschmann et al., 2015). To examine whether *C. burnetii* infection alters Tf subcellular localization, infected or uninfected HeLa cells loaded with Tf488 for 30 min were imaged by fluorescence microscopy. As expected, both infected and uninfected cells exhibited Tf488-positive vesicles dispersed throughout the cytoplasm (Figure 1). Additionally, in cells harboring large PV, prominent Tf488-positive puncta were juxtaposed with the vacuolar membrane, suggesting that *C. burnetii* may alter endocytic trafficking to recruit vesicles to the PV.

### Cells infected with *C. burnetii* exhibit more internalized Tf488

**Figure 2:**
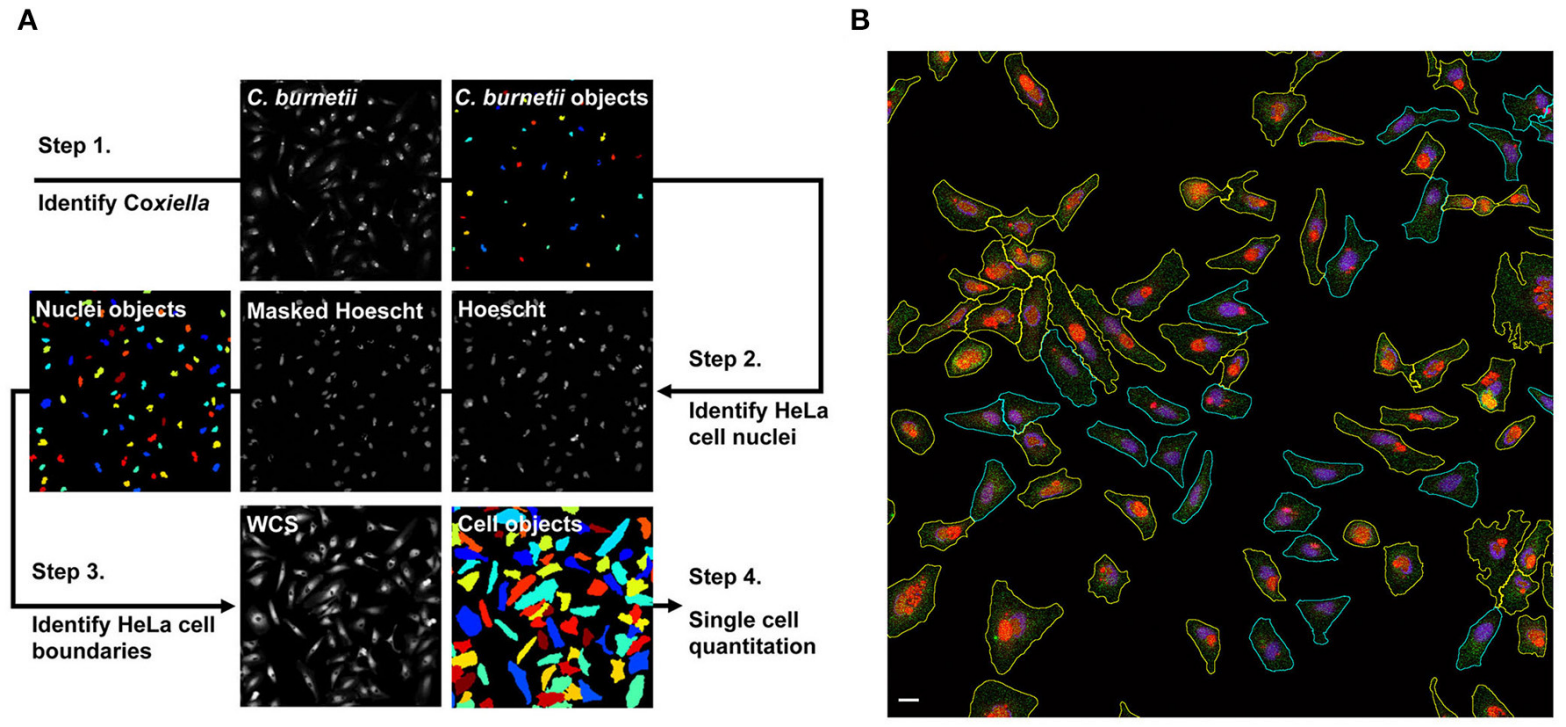
**Summary of CellProfiler image segmentation and analysis pipeline**. **(A)** Hoescht, Tf488, *C. burnetii*, and whole cell stain (WCS) channels were imported to CellProfiler as gray-scale Tiffs. In step 1, the *IdentifyPrimaryObjects* module was used to define *C. burnetii*. In step 2, the Hoescht channel was masked with *C. burnetii* objects to remove signal from bacterial nucleic acids, then the *IdentifyPrimaryObjects* module was applied to define host cell nuclei. In step 3, cell boundaries on the WCS channel were defined using the *IdentifySecondaryObjects* module to propagate from the host cell nuclei identified in step 2. In step 4, CellProfiler modules *MeasureObjectIntensity* and *MeasureObjectShapeSize* were used to quantitate multiple size, shape, and intensity parameters for each of the cell objects. **(B)** Representative fluorescence micrograph depicting segmentation and sorting of infected cells with large PV. Infected HeLa cells loaded with Tf488 (green) were stained with rabbit anti-*C. burnetii* serum and Alexa Fluor 594 conjugated anti-rabbit secondary antibody (red) and Hoescht (blue). (The green channel intensity was increased for better visibility of Tf488.) CellProfiler was used to sort infected cells (cyan outlines) by *C. burnetii* intensity per cell and to select those cells with large PV (yellow outlines). Scale bar, 20 μm.

Measurement of internalized Tf over time is commonly used to determine the rate of cellular endocytosis (Hirschmann et al., 2015). To determine if *C. burnetii* infection alters endocytic trafficking, the amount of internalized Tf488 in cell cultures infected with *C. burnetii* for 1, 3, or 5 days was compared to internalized Tf488 in uninfected cell cultures using quantitative fluorescence microscopy. Cells were incubated with Tf488 for 0–30 min, then fixed and stained for imaging. CellProfiler was used to identify cells within each image and to quantitate multiple phenotypic parameters for each cell, including the intensity of light emitted by fluorescently labeled *C. burnetii* in the Alexa Fluor 594 (AF594) channel (Figure 2A). The level of *C. burnetii* infection varied among individual cells in infected cell cultures, ranging from cells with large PV that emitted strongly in the AF594 channel, to cells lacking *C. burnetii* that emitted little AF594 signal. Due to non-specific staining by the primary antibody used to label *C. burnetii* for imaging, even uninfected cell cultures emitted some AF594 signal. To examine the amounts of Tf488 internalized by only cells harboring large PV, CellProfiler was used to select a subset of cells from infected cell cultures that emitted strongly in the AF594 channel. A threshold for AF594 signal was set in CellProfiler above the background signal emitted by uninfected cell cultures. Cells selected based on these criteria contained obvious PV (Figure 2B) and comprised ~40% of all the cells in infected cell cultures at 3 or 5 days post-infection. Further validating these selection criteria, no cells with large PV were detected in infected cell cultures at 1 day post-infection, a time point prior to exponential growth of *C. burnetii* (Coleman et al., 2004).

**Figure 3:**
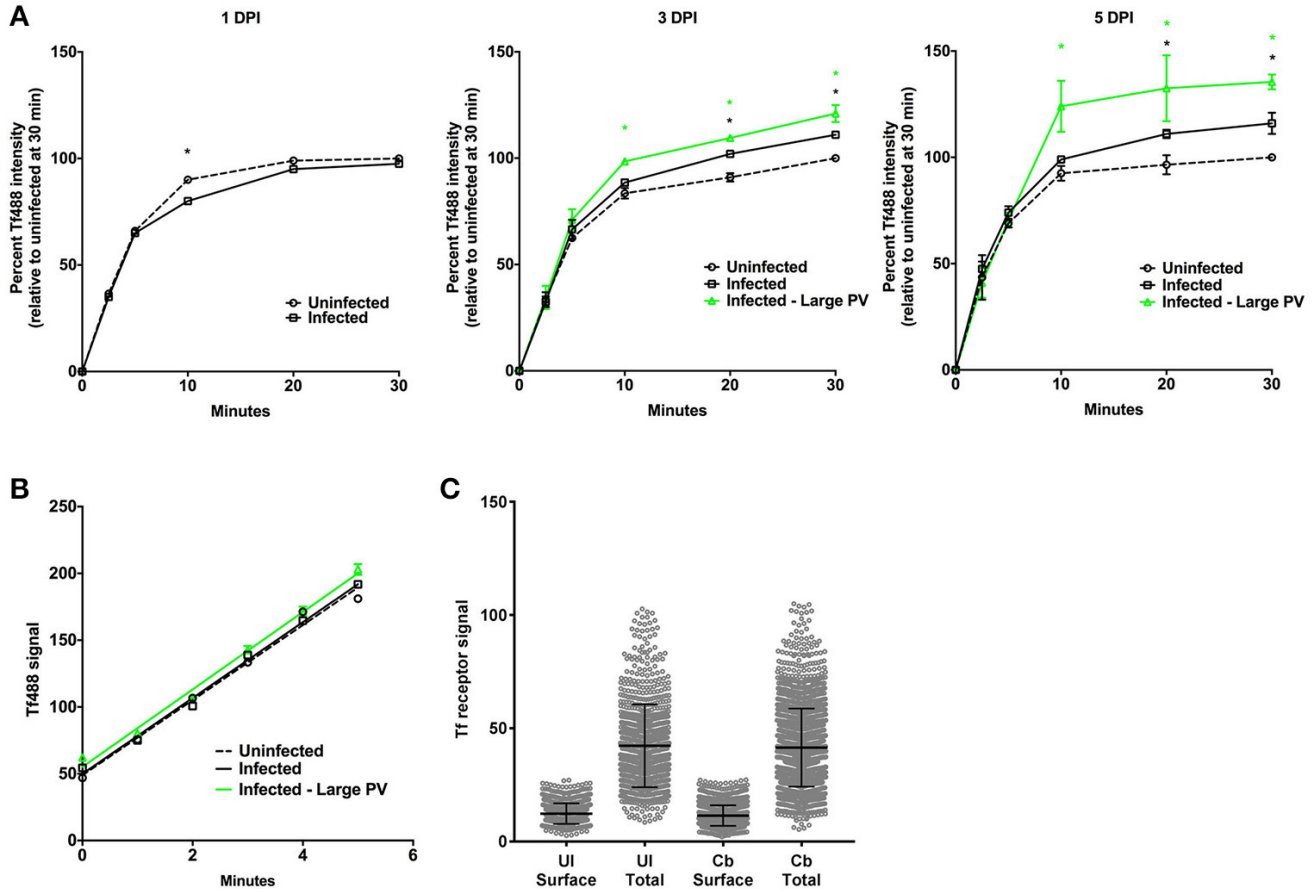
**Cells infected with *C. burnetii* exhibit more internalized Tf**. **(A)** HeLa cells infected with *C. burnetii* or left uninfected were incubated with Tf488 for the indicated times. CellProfiler was used to select only cells that emitted strong *C. burnetii* signal (Infected—large PV) from the total population of cells in infected cell cultures (Infected). The amount of intracellular Tf was measured by quantitative fluorescence microscopy. Plots depict percent Tf488 intensity calculated relative to uninfected cell cultures (Uninfected) at 0 min. A black asterisk indicates the mean Tf intensity of infected cell cultures is significantly greater than for uninfected cell cultures. A green asterisk indicates the mean Tf intensities of the subset of cells containing large PV is significantly greater than for infected or uninfected cell cultures. Statistical significance (*P* < 0.01) was determined by two-way ANOVA using Tukey's test for multiple comparisons with the mean number of cells counted per sample equal to 1038 ± 411. **(B)** Similar slopes were detected in the linear first 5 min of the Tf endocytosis assay, indicating increased internalized Tf by infected cells is not due to an increase in the rate of uptake (3 days post-infection). The plot depicts mean Tf488 signal intensity (integrated intensity per cell) with the mean number of cells counted at each time point equal to 1553 ± 178. **(C)** Increased internalized Tf by infected cells (total population) is not associated with increased surface expression of Tf receptor. Uninfected and infected cells (3 days post-infection) were fixed and stained for surface and total cellular Tf receptor. The mean number of cells counted per sample is equal to 2437 ± 582. Results are representative of 3 independent experiments and error bars indicate the standard errors from the means.

The mean Tf488 intensities per cell of infected and uninfected cell cultures, as well as for the subset of cells in infected cell cultures harboring large PV, revealed a rapid increase in the amount of internalized Tf488 over the first 10 min of the endocytosis assay, with intracellular levels of Tf488 reaching steady-state at 20–30 min (Figure 3A). Compared to uninfected cell cultures, infected cell cultures at 3 or 5 days post-infection, but not 1 day post-infection, contained significantly more intracellular Tf488 at the 20 to 30 min time points. The mean Tf488 signal emitted by infected cell cultures at 3 and 5 days post-infection was 10 and 16% greater, respectively, than for uninfected cell cultures at 30 min, with the largest increases consistently occurring with cells infected 5 days. Increases in intracellular Tf488 in response to *C. burnetii* infection were more pronounced in cells harboring large PV. Relative to uninfected cell cultures at the 30 min time point, the mean Tf488 signal emitted by cells with large PV increased 21 and 36% at 3 and 5 days post-infection, respectively. Moreover, at the 10 to 30 min time points, the Tf488 signal emitted by the subset of cells harboring large PV was statistically greater than the total emission by infected cell cultures. To determine if *C. burnetii* infection alters the rate of Tf488 internalization, fluorescence signals were measured every minute for 5 min at 3 days post-infection. The amounts of intracellular Tf488 increased linearly within this time frame with the slope of the line equaling the apparent rate of internalization. Linear regression analysis of these data indicated similar apparent rates of internalization for uninfected cells, infected cells, and infected cells containing large PV (Figure 3B). The rate of internalization is dependent on the amount of cell surface receptor (Hirschmann et al., 2015). Consistent with uptake data, no increase in surface Tf receptor was detected between infected and uninfected cells at 3 days post-infection (Figure 3C).

### Cells harboring large *C. trachomatis* inclusions have more intracellular Tf and display an increased rate of Tf internalization

**Figure 4:**
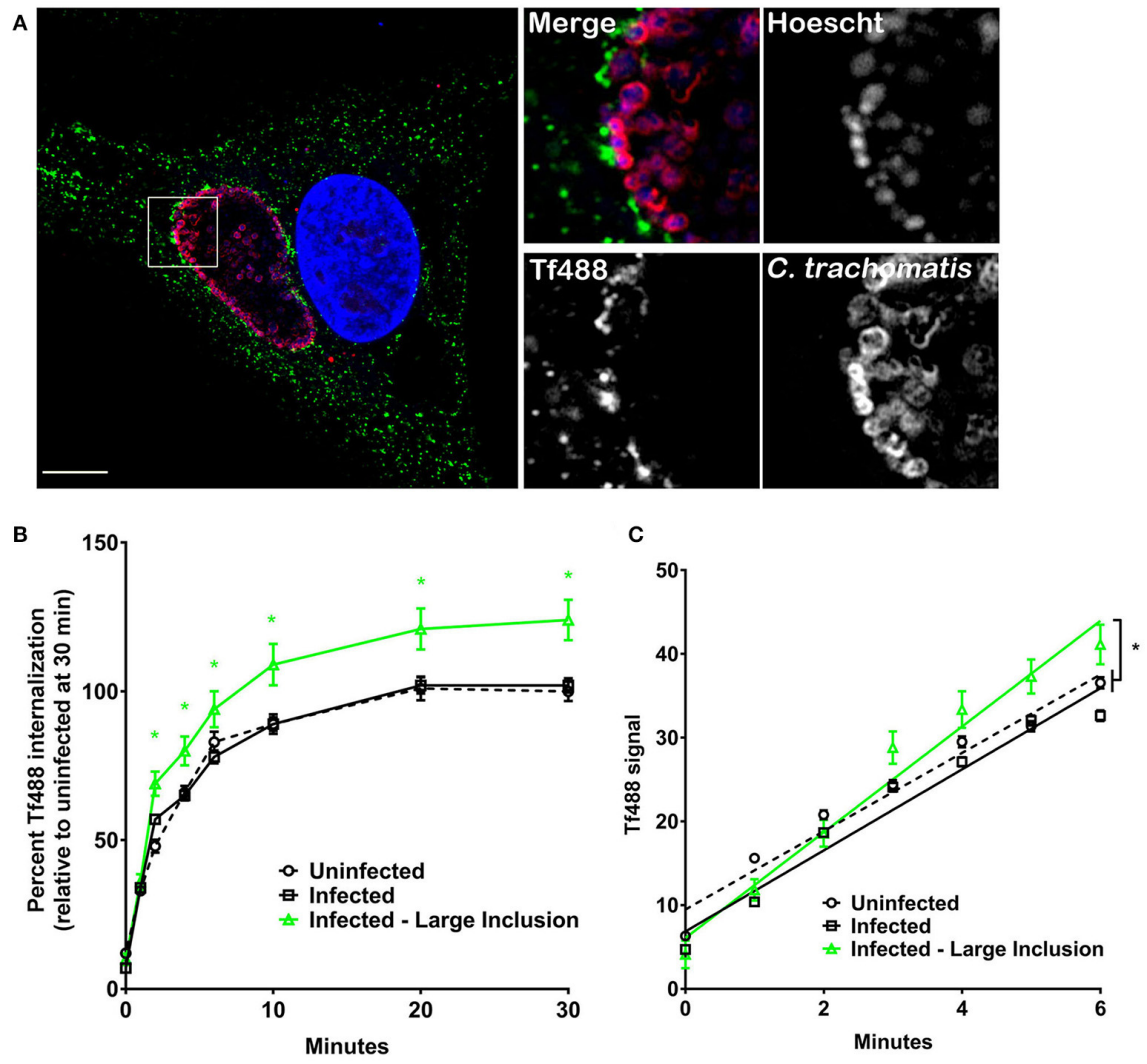
**Cells harboring large *C. trachomatis* inclusions have more intracellular Tf and display an increased rate of Tf internalization**. **(A)** Fluorescence micrograph of an infected HeLa cell showing a representative large inclusion harboring *C. trachomatis* (26 h post-infection). Cells incubated with Tf488 (green) for 30 min were fixed and stained with rabbit anti-*C. trachomatis* serum and Alexa Fluor 594 conjugated anti-rabbit secondary antibody (red). DNA (blue) was stained with Hoescht. Scale bar, 10 μm. **(B,C)** HeLa cells (26 h post-infection) infected with *C. trachomatis* or left uninfected were incubated with Tf488 for the indicated times. CellProfiler was also used to select only cells that emitted strong *C. trachomatis* signal (Infected—Large Inclusion) from the total population of cells in infected cell cultures (Infected). The amount of intracellular Tf was measured by quantitative fluorescence microscopy. **(B)** Graph depicting percent Tf488 intensity calculated relative to uninfected cell cultures (Uninfected) at 0 min. A green asterisk indicates the mean Tf intensities of the subset of cells containing large inclusions is significantly greater than for infected or uninfected cell cultures. Statistical significance (*P* < 0.005) was determined by two-way ANOVA using Tukey's test for multiple comparisons with the mean number of cells counted per sample is equal to 1062 ± 305. **(C)** Graph showing cells harboring large inclusions exhibit a greater apparent rate of Tf488 internalization as indicated by the steeper slope of the line within the linear first 6 min of the assay. The mean number of cells counted per sample is equal to 1338 ± 175. Results are representative of 3 independent experiments and error bars indicate the standard errors from the means. Statistical significance (asterisk, *P* < 0.0001) was determined by one-way ANOVA using Tukey's test for multiple comparisons.

We measured Tf internalization by HeLa cells infected with *C. trachomatis*, an intracellular bacterial pathogen that also generates a large vacuole (inclusion) in which to replicate (Figure 4A). Cells infected with *C. trachomatis* for 26 h were loaded with Tf488 and analyzed with CellProfiler as above for *C. burnetii*. During the 30 min assay, there was no statistical difference between the mean Tf488 intensities of uninfected and infected cell cultures (Figure 4B). However, infected cells containing large inclusions (~45% of all cells) exhibited significantly greater internalized Tf488 that correlated with an increase in the apparent rate of internalization relative to the uninfected cells (Figure 4C). Thus, unlike *C. burnetii* infection, altered Tf trafficking was only associated with HeLa cells containing large chlamydial inclusions and associated with an elevated rate of Tf internalization.

### Elevated intracellular Tf in *C. burnetii*-infected cells is associated with increased cellular CD63

**Figure 5:**
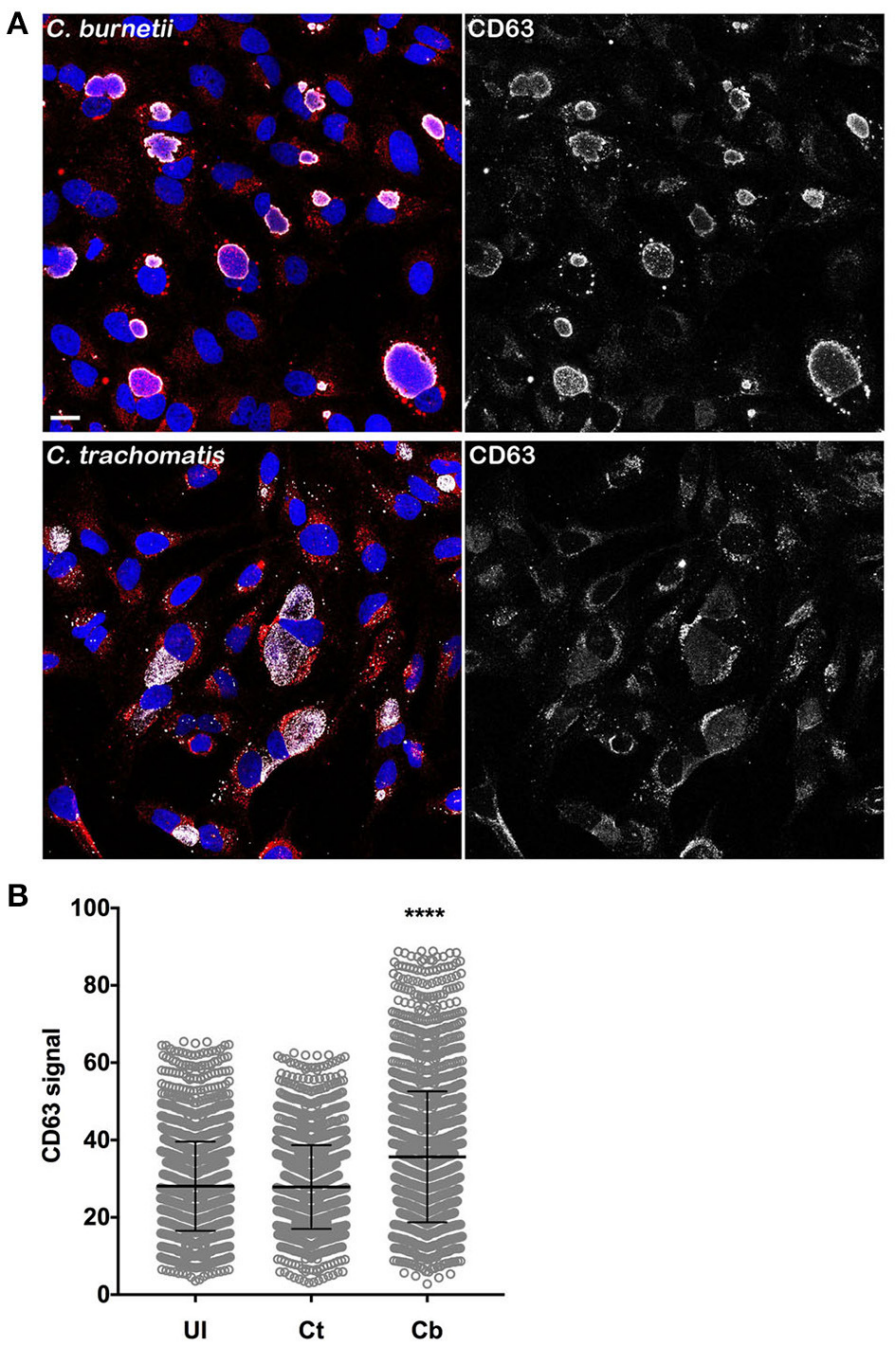
***Elevated intracellular Tf in C. burnetii-infected cells is associated with increased cellular CD63***. **(A)** HeLa cells infected for 3 days with *C. burnetii*, or 26 h with *C. trachomati*s, were stained for CD63 by immunofluorescence using anti-CD63 monoclonal antibody and Alexa Fluor 568 conjugated anti-mouse secondary antibody. Scale bar, 20 μm. **(B)** CellProfiler was used to measure the CD63 signal per cell. *C. burnetii*-infected cells contained significantly more cellular CD63 than uninfected cells. No difference was observed between uninfected and *C. trachomatis*-infected cells. Statistical significance (asterisk, *P* < 0.0001) was determined by two-way ANOVA using Tukey's test for multiple comparisons with the mean number of cells counted per sample is equal to 1894 ± 207.

To understand how *C. burnetii* infection could result in higher levels of intracellular Tf, we examined whether PV biogenesis expands the endosomal compartment. CD63 (LAMP3) is a membrane protein primarily located in late endosomes and lysosomes (Pols and Klumperman, 2009). As such, cellular CD63 content can be used as a general measure of the size of the endolysosomal system (Xinhan et al., 2011; Martina et al., 2014; Awad et al., 2015). Uninfected HeLa cells and cells infected for 3 days with *C. burnetii*, or 26 h with *C. trachomati*s, were stained for CD63 by immunofluorescence and CellProfiler used to measure the CD63 signal per cell. *C. burnetii*-infected cells contained significantly more cellular CD63 than uninfected cells (Figures 5A,B). No difference was observed between uninfected cells and cells infected with *C. trachomatis*. Thus, the increase in internalized Tf during *C. burnetii* infection correlates with increased endolysosomal content.

### *C. burnetii* infection does not alter the rate of Tf recycling and PV-associated Tf488 puncta persist after extensive recycling

**Figure 6:**
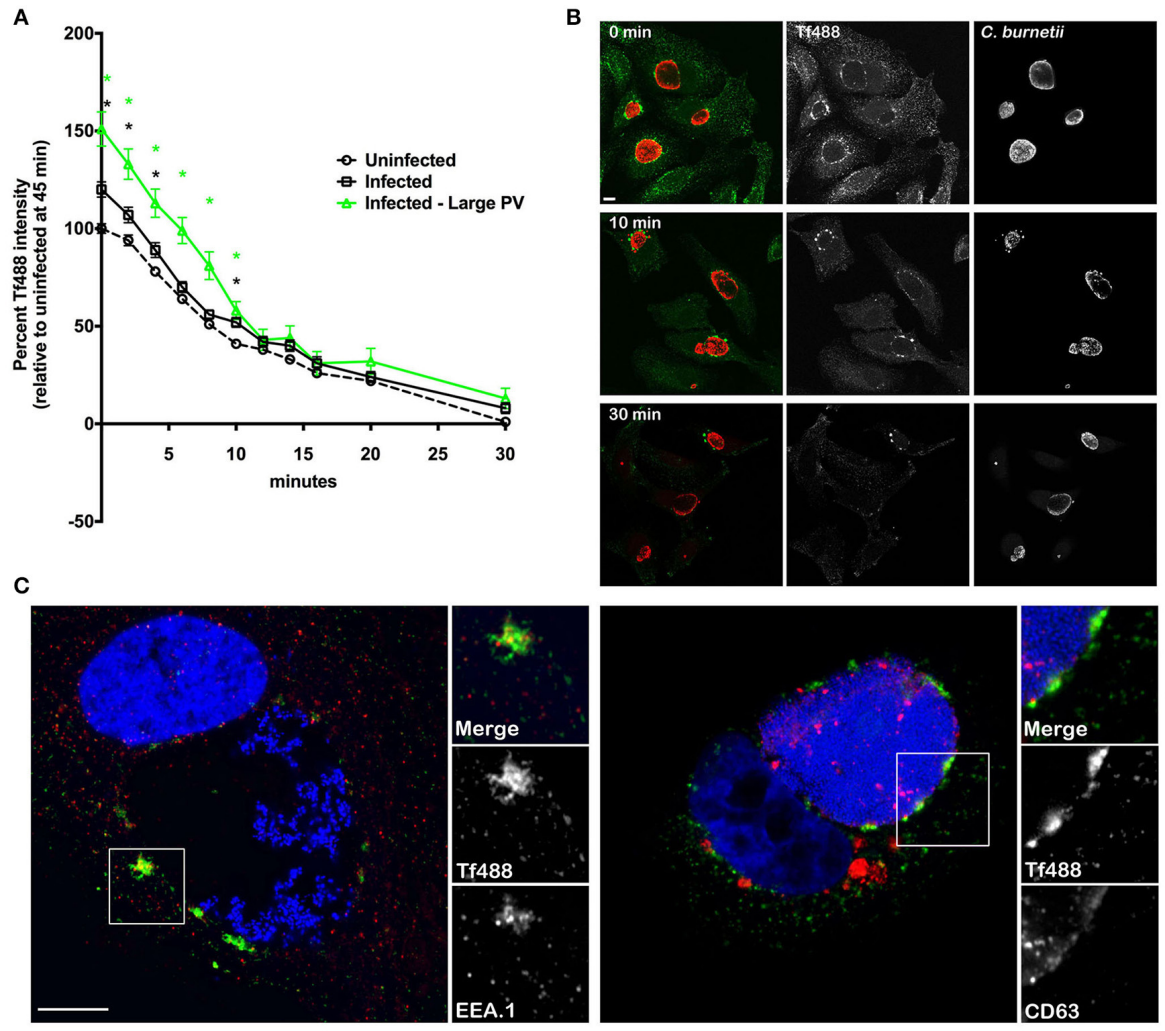
***C. burnetii* infection does not alter the rate of Tf recycling and PV-associated Tf puncta persist after extensive recycling**. **(A)** HeLa cells infected with *C. burnetii* for 3 days or left uninfected were loaded with Tf488 for 45 min and the decrease in intracellular Tf488 was measured by quantitative fluorescence microscopy. Plots depict percent Tf488 intensity calculated relative to uninfected cell cultures (Uninfected) at 0 min. CellProfiler was used to select only cells that emitted high *C. burnetii* signal (Infected—Large PV) from infected cell cultures (Infected). Statistical significance (*P* < 0.01) was determined by two-way ANOVA using Tukey's test for multiple comparisons with the mean number of cells counted per sample equal to 945 ± 301. A green asterisk indicates the mean Tf intensity of the subset of cells containing large PV is significantly greater than for uninfected cells, while a black asterisk indicates the mean Tf intensities of the subset of cells containing large PV and infected cell cultures are significantly greater than for uninfected cells. However, regression analysis of the linear first 10 min of the assay indicate the apparent rates of recycling for uninfected cells, infected cells, and infected cells containing large PV are similar. Results are representative of 3 independent experiments and error bars indicate the standard errors from the means. **(B)** Representative confocal fluorescence micrographs depicting the intracellular localization of Tf488 (green) in HeLa cells infected with *C. burnetii* (red) for 3 days, and loaded with Tf488 for 45 min, then incubated in medium lacking Tf488. After a 0, 10, or 30 min incubation, samples were fixed and immunostained with rabbit anti-*C. burnetii* serum and Alexa Fluor 594 conjugated anti-rabbit secondary antibody. PV-associated Tf puncta persist after the 30 min chase. Scale bar, 10 μm. **(C)** Tf puncta remaining in HeLa cells (5 days post-infection) after Tf488 (green) labeling and a 30 min chase were immunostained for CD63 or EEA.1 using monoclonal antibodies and Alexa Fluor 568 conjugated anti-mouse secondary antibody (red). Bacterial and host DNA (blue) were stained with Hoescht. Tf puncta co-localize with EEA.1, and to a lesser extent, CD63. Scale bar, 10 μm.

After Fe^3+^ is released into early endosomes and transported to the cytosol, Tf remains bound to Tf receptor and the complex is recycled to the cell surface (Maxfield and McGraw, 2004; Hirschmann et al., 2015). To examine whether Tf recycling is altered in response to *C. burnetii* infection, HeLa cells infected for 3 days were loaded with Tf488 for 45 min and the decrease in internalized Tf488 was measured over time. Consistent with results shown in Figure 3, the mean amount of Tf488 internalized by infected cell cultures and infected cells harboring large PV, was significantly greater than for uninfected cell cultures at the start of the recycling assay (0 min) (Figure 6A). However, regression analysis of the linear first 5 min of the assay indicated the apparent rates of recycling for uninfected cells, infected cells, and infected cells containing large PV were similar. Thus, *C. burnetii* infection increases the amount of intracellular Tf without altering the rate of endocytic recycling.

Intriguingly, at the 30 min time point, when nearly all Tf488 is recycled out of uninfected cell cultures, a small amount was detected in cell cultures infected with *C. burnetii*. To examine this phenomenon further, high magnification confocal z-stack images of infected cells were acquired throughout the Tf488 recycling assay. A time-dependent decease in internalized Tf488 was evident when comparing images of cells at the start of the assay (0 min) to cells after 10 or 30 min of recycling (Figure 6B). However, in cells with large PV, Tf488 puncta remained visible adjacent to the PV membrane when little cytoplasmic Tf488 remained (Figure 6C). Persistent Tf puncta partially co-localized with EEA.1 in vesicles adjacent to CD63-positive endosomal compartments, suggesting Tf488 follows its canonical trafficking itinerary through the endosomal compartment during *C. burnetii* infection.

## Discussion

Large size, pronounced fusogenecity, and temporal stability are characteristics of the *C. burnetii* PV that differentiate this specialized replication niche from typical phagolysosomes. Although knowledge of membrane trafficking pathways required for PV biogenesis has increased in recent years, it remains unclear how *C. burnetii* acquires materials for PV formation. Here, measurement of endocytic trafficking using fluorescently labeled Tf revealed that *C. burnetii*-infected cells have significantly more internalized Tf than uninfected cells. The greatest increases in intracellular Tf occur in cells infected with *C. burnetii* for longer periods of time and in cells harboring more bacteria, and consequently, larger PV. Increased intracellular Tf occurs without altering the apparent rates of Tf internalization or recycling. Furthermore, higher surface Tf receptor densities are not detected in infected cells. Perturbation of endocytic transport appears to occur throughout the course of *C. burnetii* PV maturation due to expansion of the endosomal compartment, as evidenced by increased cellular CD63. Further supporting this hypothesis is a recent report showing upregulation of the endocytic coat protein clathrin during the *C. burnetii* infectious process (Latomanski et al., 2016). Collectively, our data suggest establishment of a large, mature PV coincides with increased capacity for endocytic material within the endosomal system.

In the recycling assay, Tf-positive puncta persist next to the PV after extensive recycling in the absence of Tf488. The nature and function of these Tf puncta are unknown; however, co-localization of Tf with EEA.1, and to a lesser extent CD63, suggests the fluorescent probe is transiting normally between early and recycling endosomal compartments. Others have shown that under normal conditions the half-time (t_1/2_) for Tf internalization is ~2 min and the t_1/2_ of recycling to the cell surface ~10 min (Hopkins and Trowbridge, 1983; Mayor et al., 1993; Maxfield and McGraw, 2004). For proteins, such as Tf, that cycle between the surface and the interior of the cell, the slow step of recycling results in accumulation within the endocytic recycling center (Maxfield and McGraw, 2004). The enlarged endosomal compartment of *C. burnetii*-infected cells appears to accommodate greater Tf accumulation before the entry and exit pathways reach equilibrium, resulting in greater steady-state amounts of internalized Tf and longer-lived puncta adjacent to the PV. A small, undetectable change in the rate of Tf entry or exit might also contribute to greater Tf accumulation in *C. burnetii*-infected cells.

The different endocytic trafficking responses between cells infected with *C. trachomatis* or *C. burnetii* reported here are consistent with distinct mechanisms of pathogen vacuole biogenesis. The *C. burnetii* PV extensively engages the endolysosomal/autophagolysosomal pathways (Larson et al., 2016). Conversely, the chlamydial inclusion is largely disconnected from endocytic pathways, but instead fuses with exocytic vesicles derived from the Golgi apparatus (Fields and Hackstadt, 2002). The total population of cells infected with *C. trachomatis* does not display significantly greater intracellular Tf than uninfected cells, nor do they upregulate CD63. Only the subset of cells containing large inclusions exhibit increased internalized Tf, a process that correlates with an increase in the apparent rates of internalization. Therefore, *C. trachomatis* may modulate endocytic transport at later stages of inclusion maturation.

Accumulating evidence indicates *C. burnetii* modulates endocytic trafficking via the activity of T4BSS effector proteins (Larson et al., 2016). As mentioned above, CvpA and Cig57 target clathrin-mediated endocytosis, suggesting modulation of endocytic trafficking is crucial for generating the large vacuolar replication niche of *C. burnetii* that appears predominantly composed of late endosomal components (Larson et al., 2013, 2016; Latomanski et al., 2016). Expression of recombinant CvpA lacking domains that interact with clathrin and AP2 fails to rescue impaired growth of a *cvpA* mutant, a phenotype that mirrors defective PV expansion observed in response to inhibition of receptor-mediated endocytosis by knockdown of clathrin or AP2 (Larson et al., 2013). Intriguingly, ectopic expression of mCherry-tagged CvpA appears to inhibit Tf internalization (Larson et al., 2013), a result that conflicts with the increased intracellular Tf associated with *C. burneti*-infected cells reported in the current study. A possible explanation for these opposing results is that translocation of CvpA across the PV membrane by *C. burnetii* during native infection confers spatiotemporal regulation that is absent when CvpA is ectopically expressed in uninfected cells, a condition that may lead to non-specific effector activity within the cell. Indeed, spatiotemporal constraints are known to influence the activity of effectors secreted by other intracellular bacterial pathogens (Galán, 2009). An intriguing area of future investigation is the mechanism(s) of *C. burnetii* expansion of the endosomal system.

A previous report described a general increase in expression of Tf receptor by *C. burnetii*-infected J774.1 murine macrophages, but no distinction between surface and intracellular location was made (Howe and Mallavia, 1999). We do not detect differences in total or surface Tf receptor in response to *C. burnetii* infection, which is consistent with the rate data as internalization rates are dependent upon receptor availability (Hirschmann et al., 2015). Indeed, up-regulation of Tf-mediated endocytosis of Fe^3+^ may not benefit *C. burnetii*, as high concentrations of intracellular Fe^3+^ appear to inhibit *C. burnetii* growth (Briggs et al., 2008).

Tools commonly employed for examining the role of plasma membrane endocytosis, including treatment of cells with pharmacological inhibitors or ectopic expression of constitutively-active or dominant-negative proteins, can have off target effects that complicate interpretation of results (Ivanov, 2008). Additionally, many of these treatments have limited usefulness for examining cellular responses to *C. burnetii* infection because of cell toxicity issues that arise when treatments are maintained over time scales required to observe the lengthy process of PV biogenesis. Fluorescence microscopy coupled with computational analysis enables quantification of multiple cell features over large sample sizes, thereby revealing subtle changes in cell biology in the absence of potentially harsh treatments used to perturb cell physiology. Thus, high-content imaging represents a powerful approach to acquire new understanding of virulence mechanisms employed by intracellular pathogens.

## Author contributions

Conceived, designed, and performed the experiments: CL. Analyzed the data and wrote the paper: CL and RH.

## Funding

This work was supported by the Intramural Research Program of the National Institute of Allergy and Infectious Diseases, National Institutes of Health #ZAI AI000931 to RH.

### Conflict of interest statement

The authors declare that the research was conducted in the absence of any commercial or financial relationships that could be construed as a potential conflict of interest.
